# Case of Pseudo-Membranous Laryngitis, or True Croup

**Published:** 1858-07

**Authors:** J. Drake Harper

**Affiliations:** Springfield, Illinois


ARTICLE III.

### CASE OF PSEUDO-MEMBRANOUS LARYNGITIS, OR TRUE CROUP.

(EXPULSION OF FOUR ORGANISED MEMBRANES.)

BY J. DRAKE HARPER, M. D., OF SPRINGFIELD, ILLINOIS.

On Thursday the 15th day of April, I was called to see a daughter of Mr. R., aged six years, who was laboring with *true croup.* On Saturday and Sunday preceding, she complained of sore throat and some hoarseness. On Monday morning, she was out in the rain; the following night she became hoarse, difficulty of breathing, considerable fever and soreness of the throat. They gave her an emetic of comp. syr. of squills. The following morning she appeared better, and remained so until towards evening, when the breathing became very laborious; high fever, great thirst, voice sibilant and cough smothered; they gave the squills as before and bathed her feet, under the operation of the emetic. It expelled a false membrane an inch and a half long, tubulated at one end, over half an inch. After the expulsion of the membrane, the child breathed easier and supposed to be much better, run about the house, slept tolerably well the following night, and was up about the room most of the day (Wednesday), but very hoarse, same cough, voice entirely smothered. The following night the disease assumes all the former symptoms, but much more alarming, the squills were used as above stated and a warm bath. It vomited freely, and finally through a spasmodic effort at coughing and choking, a membrane was again expelled, an inch and a half long, tubulated from end to end, with blood-vessels ramifying over it. Mr. R. stated to me he could have placed it on his little finger like a *glove*, he said *it looked like a chicken's windpipe, all ridgy and rings around it.* In his anxiety to see the inside, he destroyed it.

The relief in breathing was considerable, but not so marked as before; but, strange as it may appear, the parents did not become sufficiently alarmed for the safety of the child, until the following day (Thursday), when I was summoned to see it. I arrived there about 2 o'clock P. M., found the child breathing with great difficulty, head thrown back, face flushed but bathed with perspiration; pulse 140, intermitting and rather weak; voice not above a low whisper; cough not frequent, but entirely smothered; very restless; tongue red at the point; considerable thirst; difficulty of swallowing from soreness of throat and fear of suffocation.

On examination of the throat and fauces, I found considerable exudation of plastic lymph, and congestion, and bright red inflammation of the mucous membrane of the throat and fauces, etc.; knowing the proper time for bloodletting had passed, and the third period or period of collapse had arrived, and the only hope was in the expulsion of the membrane—for this purpose I

administered an active emetic of ĉalomel, ipecac. and antimony. I waited a short time and commenced with a solution antimony, in about one hour it expelled a membrane about the same length, but only tubulated at one end a half an inch. I placed her on calomel and ipecac., to be given every two hours, and a strong solution of antimony, to be used if the breathing became difficult; and left, with orders to send for me if the case required. *In the night*, *about* 3 *o'clock a. m.*, I was again sent for. A short time before I arrived, she had expelled the fourth membrane, which I have now in my possession, tubulated at both ends, an inch and a half long. A number of my medical brethern have seen this pathological specimen of croup membrane.

The following is a partial representation of the case:

"There is generally some little expectoration, and it may be that by great efforts, some shreds of lymph may be thrown off from the larynx, with manifest relief for the time, but followed by a return of the distressing suffocation. The whole expression of the child's face, figure and posture, is one of unmitigated distress; of the agony of oppressed breathing; of the horrible dread of suffocation. It turns on every side for relief and finds none; it changes its position, lying down or sitting up, restless and anxious, as those who strive for the breath of life, and despairing as those whose efforts are in vain; awake or asleep, the distress continues; it finds no relief in the arms of its mother, no comfort in her caresses.

"From this condition the child rarely recovers. There may be occasional remissions, as I have mentioned, after the expectoration of mucus and lymph, but that is only temporary, and as the disease extends itself downwards along the bronchial tubes, all chance is excluded. The local and general distress increases; the efforts at respiration partake of a convulsive character; the passage of air through the larynx becomes more and more difficult; and after a short time, seldom above twenty-four hours, death terminates the painful scene."

Such is the graphic description given by Dr. Churchill, page 242—*Infants and Children.*

I visited my little patient on Friday afternoon. I found it beyond hope, rapidly sinking from exhaustion of the vital powers.

It had fallen into a state of stupor, and died lethargic, on Friday night.

"The younger the child the more liable it is to have the disease terminate by convulsions."—*Churchill.*

*Causes.*—"The principal causes appear to be constitutional aptitude, exposure to a cold, damp, changeable atmosphere, insufficient clothing, and epidemic miasma."

Dr. Eberle says, page 347, *Diseases of Children*—"I have no hesitation in saying that the fashion of clothing children lightly, exposing their legs, arms and necks, under the foolish notion of hardening them, is extremely favorable to the production of croup."

The Doctor has given a striking illustration of this in the case of a German settlement in America, who are in the habit of clothing their children in such a manner as to leave no part of the breast and lower portion of the neck exposed. "During a practice of six years among this class of people, I recollect having met but a single case of this affection, and this case had occurred in a family who had adopted the present universal mode of suffering the neck and superior portions of the breast to remain uncovered."

This case was one where organization and vital powers were extraordinarily good, and being raised in the country, was allowed to run wherever it desired, without regard to weather or health. Being endowed with strong vital powers, nature resisted disease, and the four membranes were expelled, which is of rare occurrence.

Dr. Churchill, page 246, in referring to Dr. Copeland on Croup, says—"That the membranous substance is detached in the advanced stages of the disease, by the secretion from the excited mucous follicles of a more fluid and less coagulable matter, which is poured out between it and the mucous coat, and as this secretion of the mucous cryptæ becomes more and more copious, the albuminous membrane is the more fully separated, and ultimately *excreted, if the vital powers of the respiratory organs and of the system are sufficient to accomplish it.*"

"Sometimes the discharge of the false membrane gives great relief; and the patient goes on recovering with a gradual, or even sudden amelioration of all the symptoms, though suppression of voice, more or less complete, for a considerable time after recovery, is not uncommon. In other cases, however, after a partial amendment, the symptoms return with all their former violence; the false membrane is *renewed*, or penetrates more deeply into the respiratory passages, and the little sufferer passes into the last and fatal stage of the disease. I have seen a relapse of this kind, after a discharge of a tube about two inches long."—*Wood's Practice of Medicine*, vol. i., page 819.

The foregoing is a faithful representation of this singular case and fatal disease, as it came under my own observation.

